# Supplementary material for: Causal interplay between lactose intolerance and gut microbiota: a combined bidirectional Mendelian randomization and in vivo validation study
Source: Front Nutr. 2026 Jun 1;13:1803337. doi: 10.3389/fnut.2026.1803337 (PMC13265576; doi:10.3389/fnut.2026.1803337)
Supplement: Supplementary file 1 [file Data_Sheet_1.zip › supplementary materials/Forward/leave one out cross validation/ebi-a-GCST90027631.finngen_R12_E4_LACTONAS.pdf]

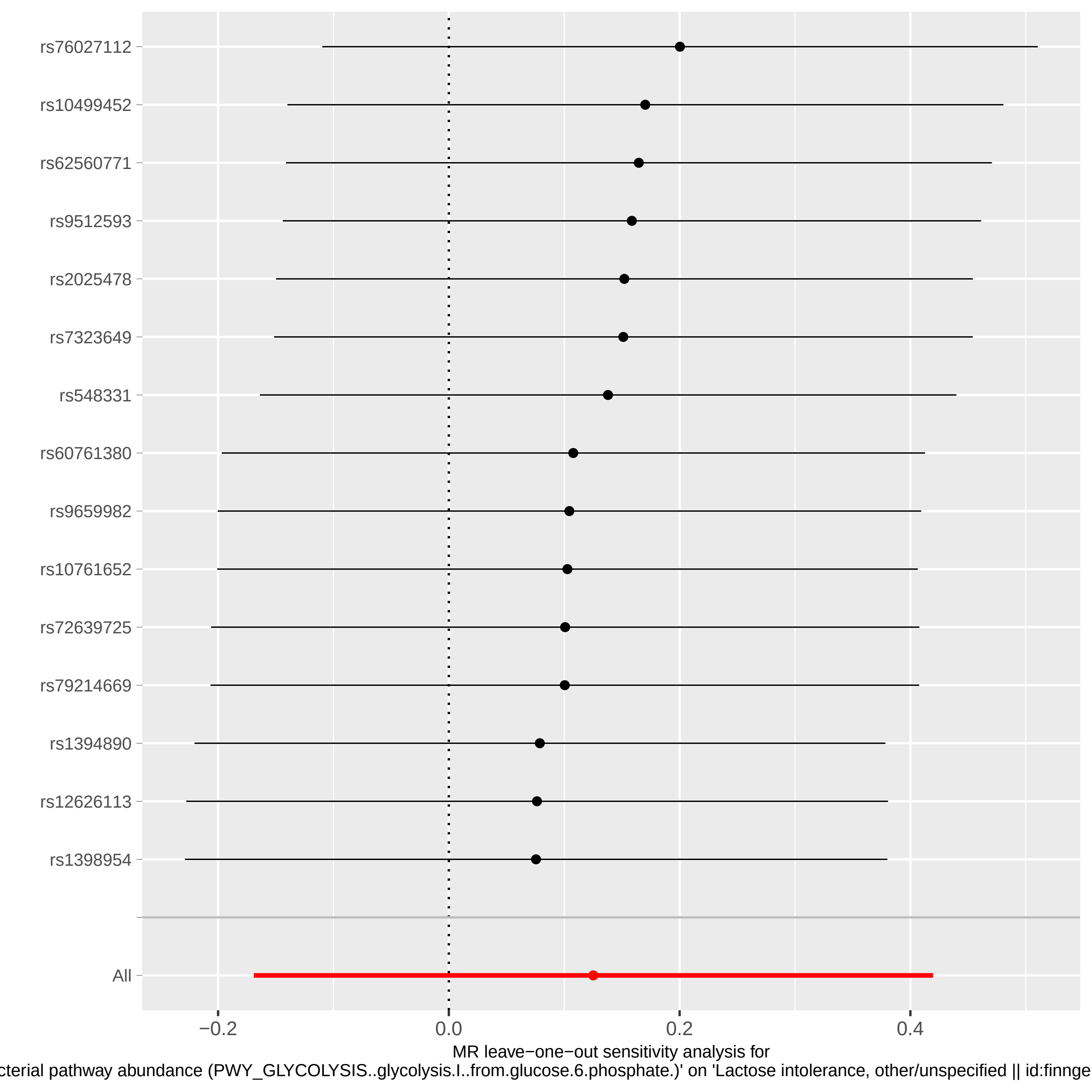

rs76027112
rs10499452
rs62560771
rs9512593
rs2025478
rs7323649
rs548331
rs60761380
rs9659982
rs10761652
rs72639725
rs79214669
rs1394890
rs12626113
rs1398954
All
−0.2
0.0
0.2
0.4
MR leave−one−out sensitivity analysis for
cterial pathway abundance (PWY_GLYCOLYSIS..glycolysis.I..from.glucose.6.phosphate.)' on 'Lactose intolerance, other/unspecified || id:finnge
